# Quantitative analysis of extracted phycobilin pigments in cyanobacteria—an assessment of spectrophotometric and spectrofluorometric methods

**DOI:** 10.1007/s10811-014-0244-3

**Published:** 2014-02-04

**Authors:** Monika Sobiechowska-Sasim, Joanna Stoń-Egiert, Alicja Kosakowska

**Affiliations:** Institute of Oceanology, Polish Academy of Sciences, P.O. box 148, Powstańców Warszawy 55, 81-712 Sopot, Poland

**Keywords:** Phycobilin concentration, Calibration, Absorbance, Fluorescence, Environmental monitoring

## Abstract

Phycobilins are an important group of pigments that through complementary chromatic adaptation optimize the light-harvesting process in phytoplankton cells, exhibiting great potential as cyanobacteria species biomarkers. In their extracted form, concentrations of these water-soluble molecules are not easily determined using the chromatographic methods well suited to solvent-soluble pigments. Insights regarding the quantitative spectroscopic analysis of extracted phycobilins also remain limited. Here, we present an in-depth study of two methods that utilize the spectral properties of phycobilins in aqueous extracts. The technical work was carried out using high-purity standards of phycocyanin, phycoerythrin, and allophycocyanin. Calibration parameters for the spectrofluorometer and spectrophotometer were established. This analysis indicated the possibility of detecting pigments in concentrations ranging from 0.001 to 10 μg cm^−3^. Fluorescence data revealed a reproducibility of 95 %. The differences in detection limits between the two methods enable the presence of phycobilins to be investigated and their amounts to be monitored from oligotrophic to eutrophic aquatic environments.

## Introduction

Adequate information on phytoplankton community structure and functional diversity is essential to protect vulnerable areas, such as densely populated coastal regions. To monitor the state of the environment, quick, cost-effective, and high-quality information is required. The utilization of diagnostic pigments for the identification and quantification of different taxonomic groups is a well-known approach. Techniques using the optical properties of active components and their signatures in cells have been studied in detail (Roy et al. 2011; Sobiechowska et al. 2010; Stoń-Egiert et al. 2010). Most of the available methods for determining phytoplankton biomass and taxonomy focus on either chlorophylls or carotenoids; only in a few instances are data for phycobilins available (Lawrenz et al. 2011; Zimba 2012).

These water-soluble protein complexes—open-chain tetrapyrroles—are the major players in light harvesting in cyanobacteria, but they are also found in red algae, cryptomonads, prochlorophytes, and glaucocytophytes (Larkum 2003). The pigment system of cyanobacteria produces only a weak chlorophyll *a* fluorescence signal. Fluorescence spectra arise mainly from photosystem II, so the fluorescence yield of phycobilins is very high, carrying a significant amount of spectral information that can be used to assess the abundance of cyanobacteria (Yentsch and Yentsch 1979). The availability of accurate concentrations for phycobilin pigments is essential for correlating reflectance with field population density; this is especially important in remote sensing. For example, the color sensors currently employed for the satellite imagery used to monitor large areas and to detect algal blooms are insufficiently sensitive to detect specific phycobilins (Woźniak et al. 2011).

In general, phycobilins absorb light in a series of overlapping peaks ranging from 450 to 660 nm. Spectral variations in phycobiliproteins are caused by site-specific chromophore–protein interactions (Bennet and Bogorad 1973; Zhao et al. 2011). The wavelengths characteristic of extracted pigments are not the same as for in vivo pigments in a membrane or bound to a protein in a phytoplankton cell, when absorption peaks are shifted to longer wavelengths (Beutler et al. 2002). All the spectroscopic methods that measure absorbance or fluorescence from phytoplankton use fixed wavelengths determined by the absorption/emission maximum.

In situ, in vivo fluorometry is a valuable tool for quickly obtaining a large quantity of spatial and temporal data for phytoplankton in the field, enabling cyanobacteria blooms to be detected in various aquatic ecosystems, especially in their coastal zones (Seppälä 2009). Typically, probes use wavelengths for fluorescence that are similar to those for the analysis of extracted pigments, but the available data obtained for phycobilin is expressed as the cell count per unit volume or as ‘relative fluorescence units’.

Although the fluorescence of a whole cyanobacterium cell has been well studied (Babichenko et al. 2000; Seppälä 2009), no standardized method is available for extracting phycobilins. While the isolation of pigments from cells still poses a challenge, existing studies focus on methods suitable for phytoplankton monocultures (Jodłowska and Latała 2010; Lawrenz et al. 2011), the pigment content of which is usually higher than that of natural phytoplankton assemblages. The extraction and purification of phycobiliproteins from algae can be a complicated and lengthy procedure, influenced by temperature, extraction time, buffer, and pH (Viskari and Colyer 2003; Lawrenz et al. 2011). Thus, current research efforts are striving to modify and optimize this process, to minimize costs and maximize yields (Zimba 2012; Horváth et al. 2013). The most efficient pigment extraction procedures combine mechanical and chemical methods that lead to protein release. They include a variety of approaches, such as buffer solution treatment (Bennet and Bogorad 1973), lysozyme digestion (Steward and Farmer 1984), asolectin-CHAPS (Viskari and Colyer 2003), freezing-thawing cycles, sonication, mechanical grinding (Lawrenz et al. 2011; Horváth et al. 2013), and capillary electrophoresis (Viskari and Colyer 2003). Accessible pigment content calculations rely principally on the equations introduced by Bennet and Bogorad (1973) and are applicable solely to absorbance data.

Our objectives in this paper were as follows: (1) to compare the analytical capabilities of two independent spectroscopic methods (spectrophotometric and fluorometric), applicable to the quantification of phycobilin pigments in aqueous extracts of phytoplankton; (2) to determine the calibration parameters enabling the detection limits of both methods to be established; and (3) to evaluate the suitability and usefulness of these methods for the analysis of phycobiliprotein concentrations in different types of samples from different ecosystems. To the best of our knowledge, such quantitative analytical procedures and the calculation of phycobilin concentrations have not been described in the literature. The rapid and precise quantitative estimate of cyanobacterial pigment concentrations in water resources may provide a timely measure of potential hazards due to the occurrence of toxic cyanobacteria.

## Material and methods

### Monocultures

The cyanobacteria cultures tested (see Table 1) include species that frequently occur in marine, coastal areas, estuaries, or lakes, and are often responsible for toxic summer blooms. Three of these species are known to contain large quantities of phycocyanin: *Microcystis aeruginosa*, *Aphanizomenon flos*-*aquae*, and *Nodularia spumigena*, and one—*Synechococcus* sp.—is rich in phycoerythrin. All the species were cultivated in the Marine Chemistry and Biochemistry Department’s laboratory, Institute of Oceanology, Polish Academy of Sciences. Strains of *N. spumigena* (ZGNS1, current No. CCNP1401) were obtained from the University of Gdańsk, Laboratory of Biochemical Ecology of Microorganisms (Gdynia, Poland), *A. flos*-*aquae* came from the University of Helsinki, while *M. aeruginosa* (PCC 7820) and *Synechococcus* sp. (PCC 7002) were obtained from the Pasteur Culture Collection of Cyanobacteria (Paris, France). Phytoplankton strains were maintained as axenic or xenic cultures, and all cultures were grown in a sterilized medium (Table 1) in 100-cm^3^ Erlenmeyer flasks. The cultures were incubated for 10 days at 22 °C ± 0.5 under continuous illumination of 10 μmol photons m^−2^ s^−1^ provided by cool white fluorescence bulbs (Kosakowska et al. 2007).

**Table 1:** Main characteristics of cyanobacteria strains chosen for studying their phycobilin and chlorophyll *a* concentrations

Cyanobacteria species	Toxins	Life form	Habitat	Growth medium	References
*Microcystis aeruginosa*	Microcystin	Spherical cells-forming colonies	Freshwater, brackish	616	Cronberg et al. 2003; Mazur-Marzec et al. 2006
*Aphanizomenon flos*-*aquae*	Saxitoxin Neosaxitoxin	Filamentous trichomes	Marine, brackish	616	
*Nodularia spumigena*	Nodularin	Filamentous trichomes	Marine, brackish, estuaries	BG11	
*Synechococcus* sp.	Heamolytic	Oval, cylindrical cells, irregular clusters picoplankton	Marine, brackish	MI	

Culture aliquots filtered in replicates of 2–10 cm^3^, depending on cell density, were concentrated on Whatman glass fiber filters of 0.7-μm nominal pore size and 25-mm diameter (GF/F). Overall, two sets of filters for each species were obtained: one for phycobilins and one for chlorophyll *a* quantification. The collected material was treated in accordance with pigment analysis protocols: Each filter was folded, wrapped in white paper and aluminum foil, frozen, and stored at −80 °C for further analysis. For the present work, two experiments involving monoculture extracts were designed: one to confirm the presence of higher concentrations of phycobilin pigments in monoculture extracts, the other to compare the results for a pure cyanobacterial extract versus those spiked with phycobilin standard(s). For this purpose, phycocyanin (PC) (4 μg cm^−3^) and phycoerythrin (PE) (5 μg cm^−3^) were added to 8 cm^3^ of *M. aeruginosa* and *Synechococcus* sp. extracts, respectively.

### Pigment extraction

The extraction protocol for phycobiliproteins was carried out according to Steward and Farmer (1984). The extraction buffer consisted of 0.25-M Trizma base (Sigma-Aldrich), hydrated 10-mM disodium EDTA (2H_2_O) (Sigma-Aldrich) and 2-mg cm^−3^ lysozyme (Merck). All three components were dissolved separately in Milli-Q distilled water (Millipore) before being combined as a single solution. According to this methodology, the initial pH 9 was acidified with hydrochloric acid, finally adjusted to precisely pH 5.5. The solution was set aside for approximately 3 h to allow it to reach equilibrium. The extraction buffer was always prepared fresh on the day of the analysis, stored in the dark at 4 °C, and was used for no longer than 48 h to prepare phycobilin standards, their dilutions and finally to extract pigments from the cyanobacterial cells.

Partially thawed filters were analyzed for their phycobiliprotein content by extracting them in buffer solution. These procedures took place in a darkened laboratory, and a combination of a gentle mechanical grinding and enzymatic (lysozyme) reaction was employed to disintegrate cell walls and improve pigment extraction efficiency. The material from each vial was vortexed (Heidolph Instruments, Germany), incubated in a dry block heat bath (Thermoleader, UniEquip) at 37 °C for 2 h, after which the samples were stored in the dark at 4 °C for 24 h. Thereafter, the samples were centrifuged (Beckman GS-6R) at 2,800×*g* for 15 min. In practice, the overall extraction time for phycobilins (from cell disintegration to pigment analysis) did not exceed 30 h. To evaluate extraction efficiency, an additional experiment consisting of two extraction cycles was carried out. The conditions that followed the isolation procedure described above were maintained throughout the course of the test.

Filters with material intended for the analysis of chlorophyll *a* and phycobilins were analyzed simultaneously. Chlorophyll *a* was extracted according to the standard procedures that recommend using 90 % acetone as a solvent by mechanical grinding and sonication (2 min, 20 kHz, Ultrasonic Homogenizer 4710 Series, Cole Parmer Instruments) in the dark at 4 °C for 2 h. The extract was centrifuged (20 min, 5 °C, 3,210×*g*, Beckman, GS-6R) to remove the filters and cell debris.

In our study, to ensure applicability of this procedure to the routine analysis of phycobilins, the aspect of multiple extractions was neglected. Ultimately, a single cycle of extraction was applied to all samples. Pigment concentrations were assessed using the equations by Jeffrey and Humphrey (1975) for chlorophyll *a* (chl *a*) and by Mantoura and Repeta (1997) for phycobilins.

### Phycobilin standards—calibration and dilution series

Phycobilin standards (ProZyme Inc., USA) were used to conduct regular calibration experiments (*n* = 5) with replicates (*n* = 3) for each of the two analytical techniques. Detailed information regarding the purchased reference material is summarized in Table 2.

**Table 2:** Summary of information provided by the certificate of analysis for phycobiliproteins (ProZyme, Inc.)

Phycobiliprotein	Code	MW (kDa)	Protein subunits	Abs_max_ (nm)	Em_max_ (nm)	Purity		Extinc. coeff. *E*_*λ*,max_^1*%*^	Origin
						A_*λ*max_/A_280_^a^	A_620_/A_566_^b^		
C-Phycocyanin	PB11	232	(αβ)_6_	620	647	4.68	n.a.	70	*Spirulina platensis*
R-Phycoerythrin	PB31	240	(αβ)_6_γ	496–566	576	5.61	<0.005	82	Red algae
Allophycocyanin	PB20	104	(αβ)_3_	648–652	660	5.3	n.a.	73	*Spirulina platensis*

*n.a*. not available

^a^Indicative of the purity of the preparation with respect to most forms of contaminating protein. Absorbance at 280 nm in these preparations is primarily due to aromatic amino acids, and this is roughly proportional to the overall concentration of protein in solution, including C-PC, R-PE, and Allo-PC. The respective absorbances at 620, 566, and 652 nm reflect only concentration

^b^Rough indicator of the level of contamination with R-PC

The pigments—5 mg of PC (PhycoPro™ C-Phycocyanin), 5 mg of APC (PhycoPro™ Allophycocyanin), and 10 mg of PE (PhycoPro™ R-Phycoerythrin)—were purchased in two lots (in 2010 and 2012) at respective concentrations of 21.2 and 25.1, 22.1 and 23.8, and 20.0 mg cm^−3^. The solutions were supplied as suspensions in 60 % ammonium sulfate, 50-mM potassium phosphate (pH 7.0) and stored in the dark at 4 °C, as recommended by the supplier.

Highly concentrated single solutions were used to estimate the maximum concentration above which instrument drift would occur. Dilution series were prepared to evaluate calibration linearity and the minimum detectable quantity of a substance. Stock solutions of single pigments and dilution series were prepared using appropriate amounts of each protein and extraction buffer as a solvent. Calibration curves were obtained for PC, PE, and APC using dilution series (from 6 to 13 levels) prepared in triplicate for each pigment. The concentration of the solutions ranged from 1 to 10 μg cm^−3^for the absorbance measurements (Spectrophotometer UV/Vis Hitachi U-2800), from 0.001 to 1 μg cm^−3^ for PC and APC, and from 0.001 to 0.5 μg cm^−3^ for PE for the fluorometric measurements (Cary Eclipse, Varian, Agilent Technologies). Calibration was repeated several times for each pigment, using two sets of phycobilin standards. The number of repetitions (from *n* = 18 to *n* = 53) yielded a low scatter of apparatus response data, which is reflected by the low standard deviations of <4.9 % (discussed in “Results and discussion”).

### Absorbance and fluorescence measurements

The calibration parameters, i.e., response factor and detection limits, were determined for the two instruments. Analyses were carried out using a double-beam spectrometer (Spectrophotometer UV/Vis Hitachi U-2800) and a single-beam, xenon lamp fluorescence spectrophotometer (Cary Eclipse, Varian, Agilent Technologies). Continuous spectra of absorbance (400–800 nm) and fluorescence emission readings (500–700 nm) were collected using 5- and 1-cm glass and quartz cuvettes, respectively. Absorbance readings at 750 nm were used for background corrections. The emission intensity signal for the extraction buffer was subtracted from the values obtained for each sample. Emission spectra with a resolution of 2 nm were obtained for every 5 nm of excitation. The calibration analysis was carried out for three settings of the excitation signal and controlled by varying the intensity of the xenon lamp, set at 600, 800, and 1000 V. The total run time for these settings would usually be about 15 min, and the calibration was carried out at ambient temperature. The extraction buffer and 90 % acetone were used as references for phycobilins and chlorophyll *a*, respectively.

## Results and discussion

### Spectral characteristics of phycobilins

**Fig. 1:**
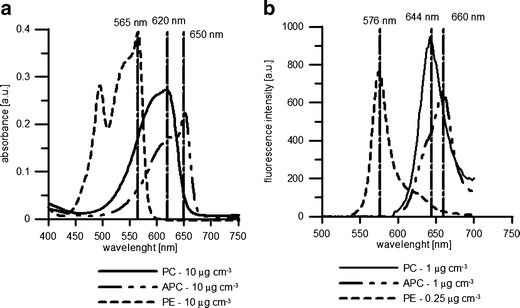
Examples of absorption (**a**) and fluorescence (**b**) spectra of phycobilin pigment standards obtained for known pigment standard concentrations. Fluorescence emission spectra of PC obtained for excitation at 590 nm, APC (600 nm), and PE (530 nm) at a lamp intensity set to 800. The marked wavelengths correspond to the maximum values of **a** absorbance and **b** emission

The spectral characteristics of the target compounds were assessed in the course of a series of experiments. Examples of the absorbance and emission spectra of the phycobiliprotein standard (PC, APC, and PE) solutions are shown in Fig. 1. The spectrophotometric and spectrofluorometric signatures of single phycobilin pigments differ in their shape, *λ*_max_ number, and position. In the absorbance spectra, PC exhibits one maximum (at 620 nm), whereas there are two peaks for PE (at 495 and 565 nm) and for APC (at 615 and 650 nm). The emission spectra displayed single peaks for PC, PE, and APC at 644, 576, and 660 nm, respectively. The spectra are in compliance with the data sheet included in the certificate of analysis, provided by the pigment standards supplier (ProZyme, Inc.).

Based on the information derived from spectra, the maximum absorbance wavelength (*λ*_max_) and the maximum emission wavelength (*λ*_em_) for a specific excitation wavelength (*λ*_ex_) for single compounds were chosen for further analysis. Thus, the following wavelengths were selected for the spectrophotometric measurements: 620 nm for PC, 650 nm for APC, and 565 nm for PE. For the fluorescence measurements, PC was investigated by exciting the molecule at 590 nm (maximum of emission *λ*_em_ = 644 nm), APC emission spectra were obtained from excitation wavelengths set at 600 nm (*λ*_em_ = 660 nm), and PE emission spectra at 576 nm were obtained from excitation at 530 nm.

### Calibration parameters and detection capabilities

**Fig. 2:**
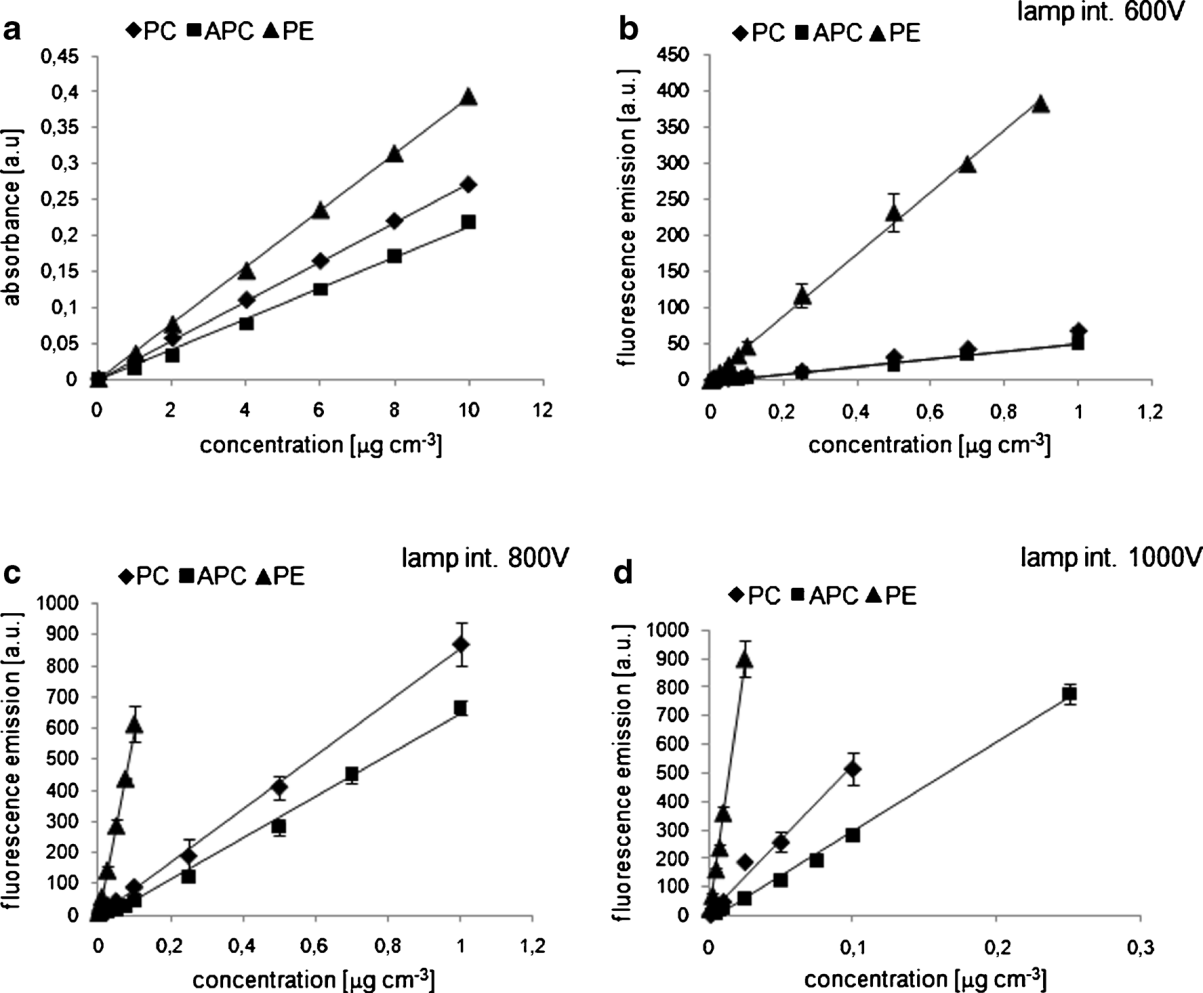
Calibration curves of phycobiliprotein standards: phycocyanin (*diamonds*), allophycocyanin (*squares*), and phycoerythrin (*triangles*) obtained for **a** spectroscopic measurements (Spectrophotometer UV/Vis, Hitachi U-2800) and **b**–**d** spectrofluorometric measurements, Fluorescence Spectrophotometer (Cary Eclipse, Varian, Agilent Technologies) for different lamp intensity settings: **b** 600 V, **c** 800 V, and **d** 1000 V. The characteristic wavelengths for which instruments gave the illustrated responses are specified in Table 3

The series of experiments and information gained regarding phycobilin concentration ranges generated multiple-point calibration curves for both measuring techniques (Fig. 2). Based on the equations obtained, calibration coefficients such as response factors, detection limits, method sensitivity, and analytical precision for the target compounds were established for different measuring conditions (Table 3). Knowledge of response factors is necessary for determining the concentrations of target compounds using the external standardization equation method (Mantoura and Repeta 1997). Values of coefficients *f*_*a*_ correspond to the slope of the linear function where concentration is plotted against instrument response. Curves were obtained in a series of experiments carried out from 2010 to 2013; the amount of calibration data points was thus substantial. The calibration curves yielded satisfactory correlation coefficients (*R* ^2^), between 0.985 and 0.999. The use of different light intensity settings enabled the concentration to be specified with coefficients *f*_*a*_ changing by one order of magnitude. The response factors for phycocyanin and allophycocyanin—compounds with a similar chemical structure and similar shape of fluorescence spectrum—were comparable for different lamp intensity settings (see Table 3).

**Table 3:** Mean response factors (*f*_*a*_) and determination coefficients (*R* ^2^) obtained for phycobilin standards (ProZyme, Inc.) dilution series

Spectrophotometer						
Symbol of phycobilin	*λ*_max_ (nm)	Number of data (*n*)	Response factor *f*_*a*_ μg cm^−3^ · [abs]^−1^		Determination coefficient (*R* ^2^)	Detection limit (μg cm^−3^)
			Symbol	Value		
PC	620	18	*f*_*a*,*PC*,*abs*_	1.83E + 02	0.999	1.00
APC	650	18	*f*_*a*,*APC*,*abs*_	2.36E + 02	0.994	1.00
PE	565	18	*f*_*a*,*PE*,*abs*_	1.27E + 02	0.999	1.00
Fluorescence spectroscopy						
Symbol of phycobilin	Lamp intensity (V)	Number of data (*n*)	Response factor *f*_*a*_ μg cm^−3^ · [a.u.]^−1^		Determination coefficient (*R* ^2^)	Detection limit (μg cm^−3^)
			Symbol	Value		
PC *λ*_ex_ = 590 nm *λ*_em_ = 644 nm	600	53	*f*_*a*,*PC*,*600*_	1.521E − 02	0.994	0.0075
	800	52	*f*_*a*,*PC*,*800*_	1.170E − 03	0.998	0.0075
	1000	42	*f*_*a*,*PC*,*1*,*000*_	1.895E − 04	0.985	0.001
APC *λ*_ex_ = 600 nm *λ*_em_ = 660 nm	600	34	*f*_*a*,*APC*,*600*_	1.991E − 02	0.991	0.01
	800	34	*f*_*a*,*APC*,*800*_	1.569E − 03	0.989	0.01
	1000	25	*f*_*a*,*APC*,*1*,*000*_	3.318E − 04	0.994	0.005
PE *λ*_ex_ = 530 nm *λ*_em_ = 576 nm	600	47	*f*_*a*,*PE*,*600*_	2.307E − 03	0.998	0.001
	800	31	*f*_*a*,*PE*,*800*_	1.671E − 04	0.998	0.001
	1000	20	*f*_*a*,*PE*,*1*,*000*_	2.808E − 05	0.997	0.001

Phycoerythrin exhibited greater sensitivity with the fluorescence technique compared to the other phycobiliproteins analyzed in this study, which was reflected by the lower detection limits obtained under the same measurement conditions (the same lamp intensity settings), and the 6–12 times smaller values of the calibration curve slopes. Overall, the concentration range for the two analytical methods used in our study revealed a difference in sensitivity of five orders of magnitude: The minimum detection limit for the spectrofluorometer appears to overlap the quantitation maximum for the spectrophotometer. This analysis indicates that phycobilins present in amounts from 0.001 to 10 μg cm^−3^ are potentially detectable, enabling the occurrence of these pigments to be investigated in different ecosystems where microorganism cells contain trace amounts of phycobiliproteins.

**Fig. 3:**
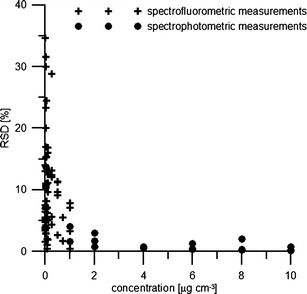
Relative standard deviation (RSD) calculated for absorbance and fluorescence data for selected dilution series of phycobilin standards

As shown in Fig. 3, the relative standard deviation (RSD) obtained for the separate dilution series of phycobilin standards ranged from 0.5 to 34.7 % for the absorbance data (*n* = 18) and from 0.15 to 4 % for the fluorescence data (*n* = 75). The mean RSD was 9.6 % (±7.3 %) for the fluorescence readings and 1.1 % (±1.1 %) for the spectroscopic data. The highest RSDs (>20 %, *n* = 7) are related to the lowest concentrations (from 0.0075 to 0.05 μg cm^−3^), whereas RSD < 10 % (mean = 5.1 ± 2.4 %) was calculated for more than 50 % of the data points obtained (*n* = 45).

### Reproducibility

**Fig. 4:**
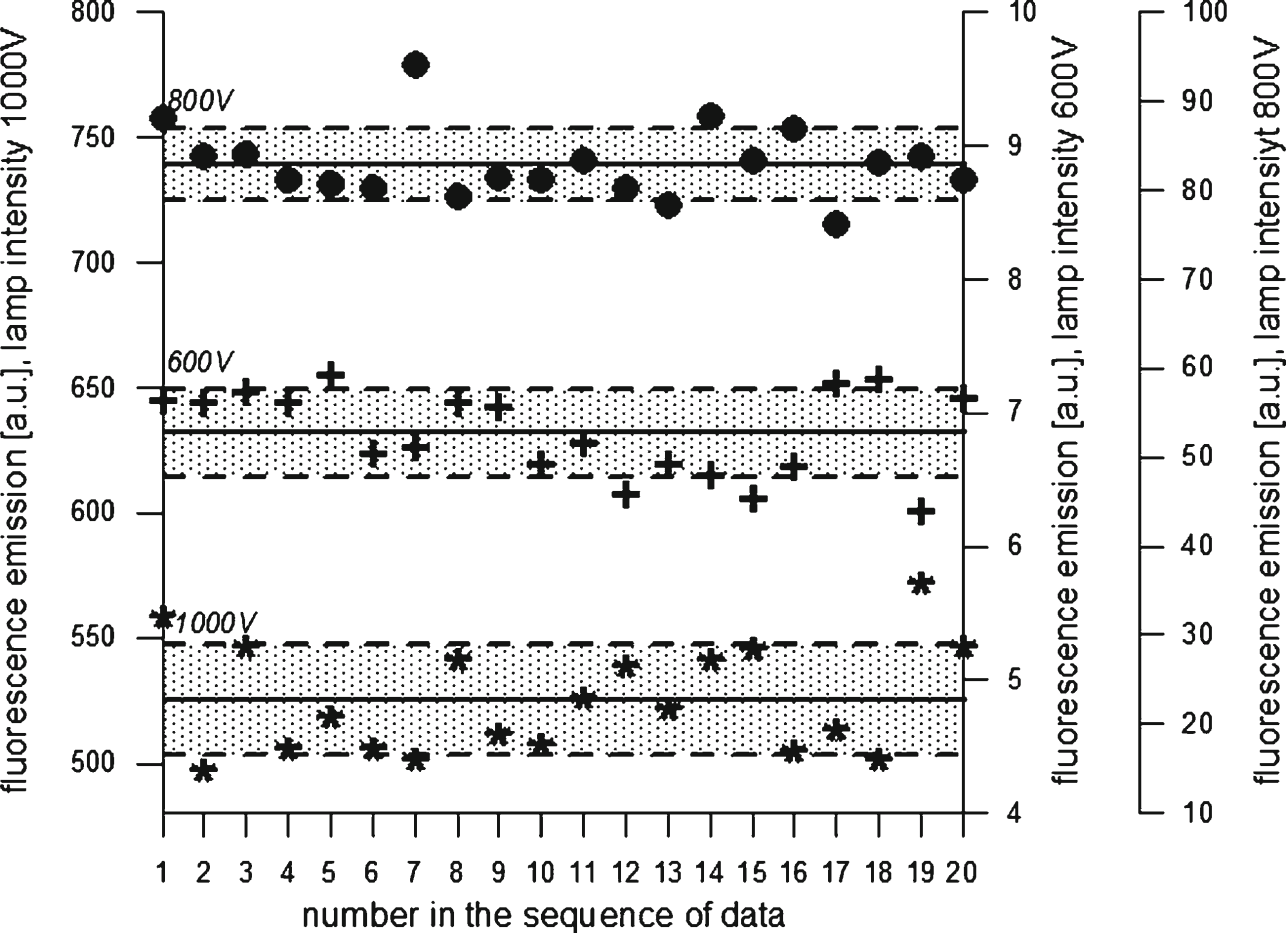
Series of fluorescence emission values obtained at λ_em_ = 644 nm for a standard solution of PC (concentration 0.1 μg cm^−3^). The fluorescence spectrometer was set to different incident light intensities (λ_ex_ = 590 nm): 600, 800, and 1000 V. *Solid line* mean value for a sequence, *dotted line* range of divergence from the mean value

The sensitivity and analytical precision of the method were specified on the basis of a series of measurements (*n* = 20) carried out at selected light excitation and emission intensities. The reproducibility of the instrument response (here, the fluorescence emission intensity) obtained for one of the pigment standards (PC) at different light intensities is presented in Fig. 4. A certain concentration (0.1 μg cm^−3^) of analyte was matched in such a way that the intensity of the instrument response to excitation achieved at 600, 800, and 1000 V would not exceed the detector’s range of sensitivity. The response intensities for *λ* = 644 nm ranged from a mean value of 6.8 ± 0.3 at 600 V to 83.0 ± 4.0 at 800 V and 525.2 ± 21.8 for 1000 V. The RSD calculated for these observations was the lowest (4.14 %) for the intensity of 1000 V; the values obtained for 800 and 600 V were at a similar level—4.9 and 4.8 %, respectively. Based on the number of observations for a PC standard solution, the average precision error of the fluorometric method was 4.6 %.

### Temporal stability of phycobilins

**Fig. 5:**
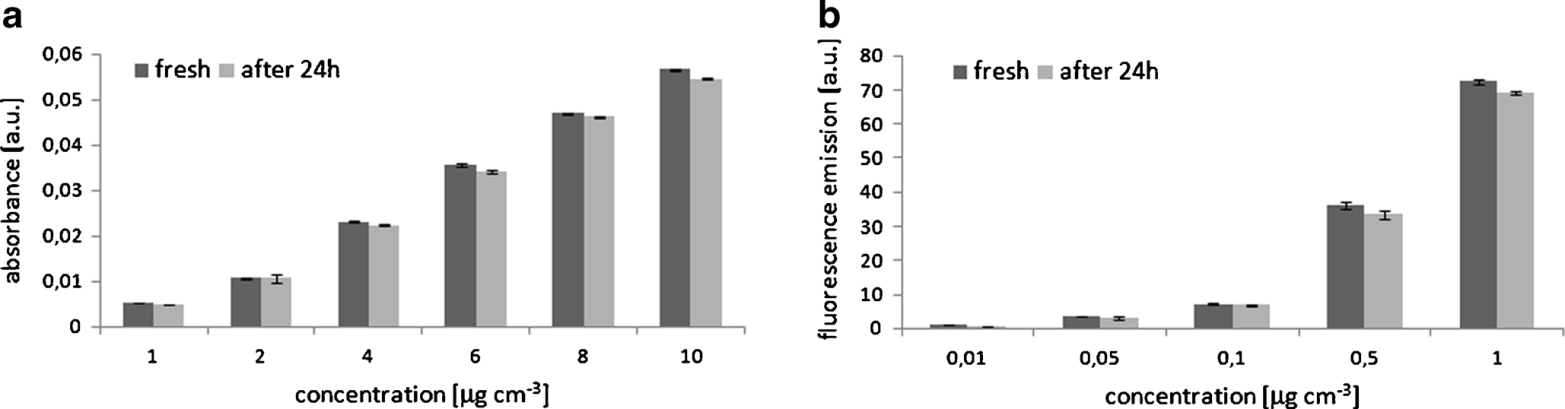
Average temporal changes in **a** absorbance and **b** fluorescence response values (lamp settings 600 V) for PC (*n* = 3). *Fresh* means measured 2 h after the solutions for extraction were prepared

During calibration, the temporal variability of the fluorescence response for standard solutions of PC, APC, and PE was also examined. We compared the results obtained 2 and 24 h following the preparation of the standard extracts (Fig. 5). There was a slight decrease in emission intensity with time. The decline in pigment concentration for PC ranged from 4.3 to 13.4 % (mean 8.8 ± 4.25 %), for PE from 0.5 to 5 % (mean 3.1 ± 3.15 %), and for APC from 13.2 to 15 % (mean 14.2 ± 0.95 %). The highest variability seemed to go hand in hand with the lowest concentration.

For the absorbance measurements, only a slight decrease was observed after 24 h relative to the absorbance of freshly prepared solutions: for PC from 0.03 to 9.2 % (3.4 ± 2.9 %) (Fig. 5a), for APC from 0 to 8.6 % (mean 2.4 ± 3.5 %), and for PE from 0 to 1 % (mean 0.4 ± 0.5 %).

An interesting observation was made concerning the extraction buffer and its stability over time. Compared to the initial conditions (immediately after the solution was prepared), there was an approximately twofold increase in emission intensity 2–3 h later. After this dynamic change, the signal from the extraction solution remained stable for at least 1 day. The inference from this is that the extraction buffer should be prepared fresh but not used right away. We recommend a period of approximately 3 h to allow the reaction to reach equilibrium. Since a spectrophotometer is known to give a less intensive response than a fluorometer, no such observation regarding the extraction solution was made in the case of absorbance.

### Phycobiliproteins in monocultures

**Fig. 6:**
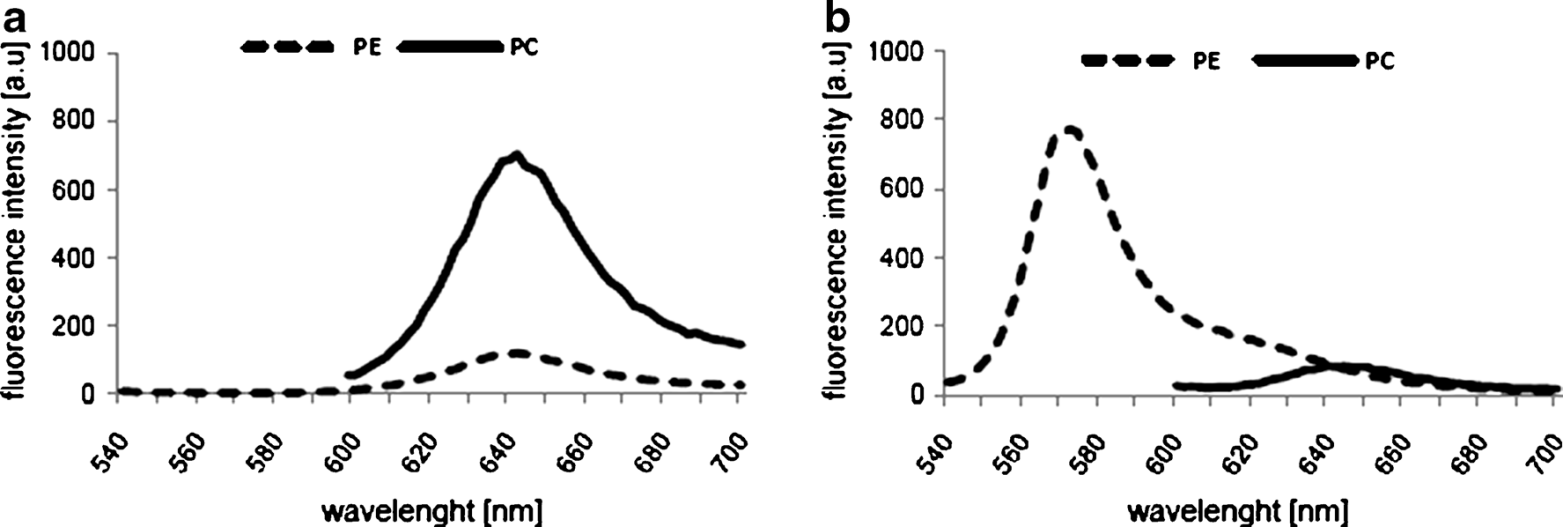
Emission spectra obtained for selected monoculture extracts. **a** *Microcystis aeruginosa*. **b** *Synechococcus* sp.

**Fig. 7:**
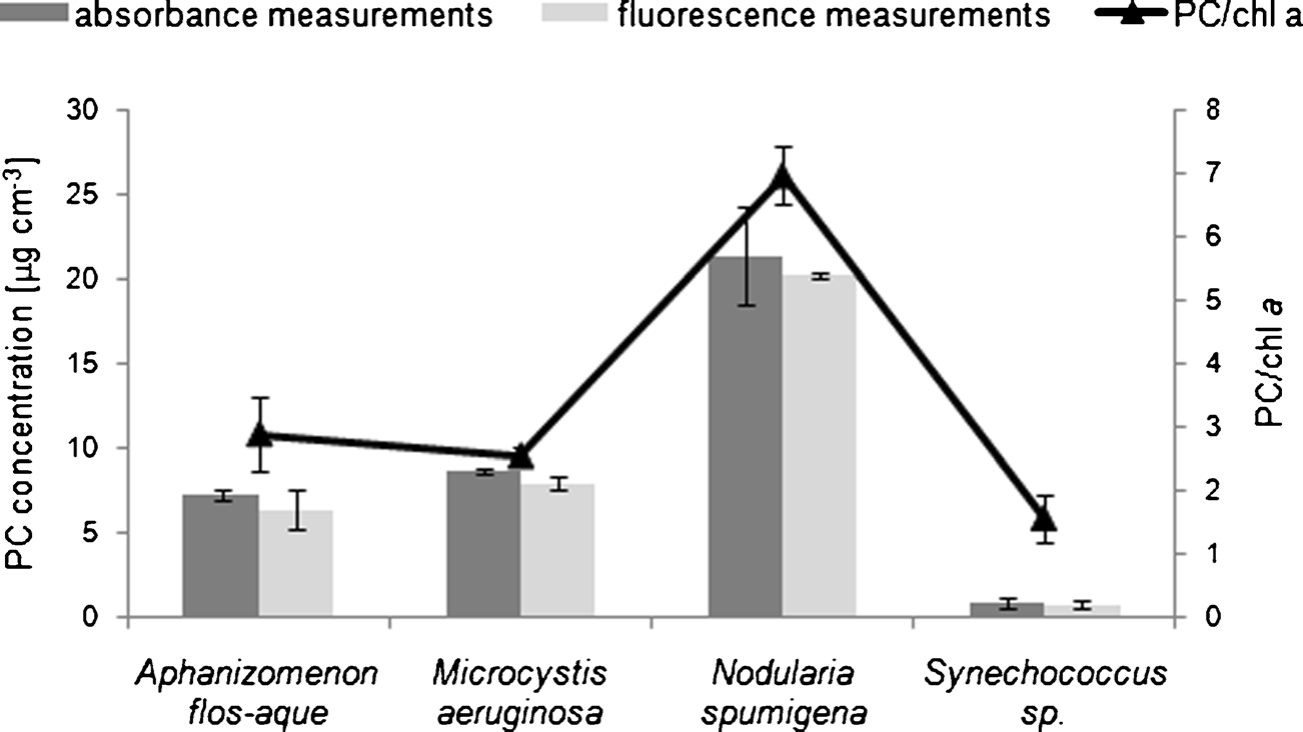
Phycocyanin concentration (μg cm^−3^) and relative PC/chl *a* ratios determined for cyanobacteria monoculture extracts. Comparison of data assessed using two analytical techniques: spectrophotometry (*blue*) and spectrofluorometry (*red*)

The suitability of the two analytical methods for determining the extracted phycobilins was examined. The pigment content in cells was assessed in four species of cyanobacteria (details in the “Pigment extraction” section). The improvement in extraction efficiency attributable to re-extraction, suggested by Horváth et al. (2013), was examined for two coccoid cyanobacteria cultures: *Synechococcus* sp. and *M. aeruginosa*. A second round of pigment extraction from cells yielded on average an additional 10.5 % of PE (*n* = 6) and 6.4 % of PC (*n* = 6) for PE-rich species and 10.4 % of PC (*n* = 6) for *M. aeruginosa*. The results are comparable with the values presented by other authors (Zimba 2012). The coefficient of variation for replicates was 20 % for PC (for both cultures) and 11 % for PE (*Synechococcus* sp.). Qualitatively, the phycobilins found in extracts of cultures matched the characteristics of the pigments (Seppälä 2009; Jodłowska and Latała 2010): PC was found in all extracts, occurring more abundantly than PE in three of the four species analyzed, giving a strong signal and a characteristic reddish colouration in *Synechococcus* sp.; APC was not found (Figs. 6 and 7).

The phycobilin concentration was calculated using two external calibration equations that gave comparable results. The difference of 8.1 % (±7.7 %) between the absorbance- and fluorescence-based measurements obtained for PC indicates an overall high accuracy. The PC concentration in the material analyzed ranged from 0.79 μg cm^−3^ for *Synechococcus* sp. to 20.22 μg cm^−3^ for *N. spumigena*. Figure 7 illustrates the PC concentrations obtained with the two analytical methods. The relative pigment content in cells varied from 1.6 to 7 for PC, but was 2.1 for PE, which was identified only in red *Synechococcus* sp. The high phycobilin/chl *a* ratios confirm that accessory pigments as photosynthetic antennae in photosynthetic apparatus of tested species, as demonstrated earlier by Beutler et al. (2003). Phycobilins (chromophores) are known to be present at higher concentrations than chlorophylls in many cyanobacteria species and red algae (Nobel 2009).

In nature, allophycocyanin is present at significantly lower levels than phycocyanin or phycoerythrin, accounting on a weight basis for approximately 10 % of the total cellular phycobiliprotein. The APC absorption maximum in whole cells or a crude extract is largely masked by the much greater absorbance of PC (Ting et al. 2002). An approach such as deconvolution may be a possible means of overcoming the method selectivity problem (MacColl 1991).

**Fig. 8:**
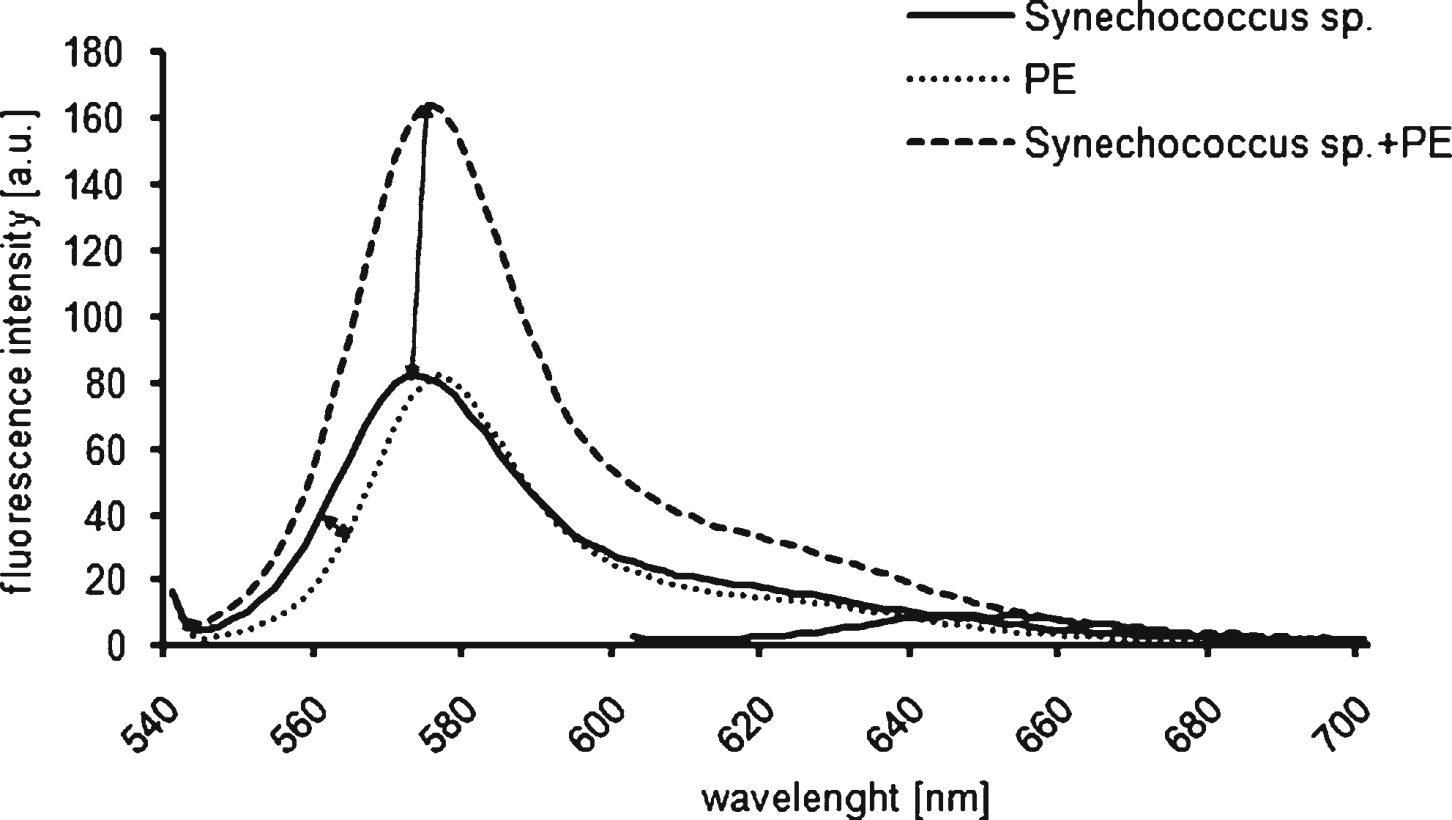
Emission spectra obtained for a PE-rich cyanobacteria monoculture

Monoculture extracts spiked with phycobilin standards revealed some interesting yet unexpected insights (Fig. 8). Firstly, the spectrum obtained from a standard solution of specific pigments differs slightly from that obtained for a monoculture extract, naturally containing pigments as well as other cellular components (e.g., different proteins) that can alter the shape of the spectrum. Secondly, spectrum height (the signal intensity) reflects the quantity of analyte and increases proportionally, which is especially evident in the case of a cyanobacteria extract spiked with a standard pigment solution. Spectrophotometry was not sensitive enough to produce an absorbance signal falling within our detection limit. On the other hand, we were able to apply spectrofluorometry to quantify phycobilin pigments extracted from phytoplankton cells.

## Conclusions

In this study, to assess the quantitative analysis of phycobilins, we verified and confirmed the poorer sensitivity of spectrophotometric methods compared with spectrofluorometry. We suggest that the former approach would be more appropriate for highly concentrated, monoculture extracts, whereas phycobilins extracted from natural waters can be identified spectrofluorometrically. Nonetheless, all the necessary steps in the analytical process should be followed in order to obtain qualitatively and quantitatively reliable methods, and that includes careful consideration of particular study needs and priorities. The determination of extracted phycobilin pigments and their application as additional biomarkers is a promising approach for the routine monitoring of cyanobacteria in coastal, densely inhabited, and less-populated aquatic environments.
